# Combined psychosocial work factors and risk of long-term sickness absence in the general working population: Prospective cohort with register follow-up among 69 371 workers

**DOI:** 10.5271/sjweh.4035

**Published:** 2022-10-01

**Authors:** Lars L Andersen, Jonas Vinstrup, Sannie V Thorsen, Jacob Pedersen, Emil Sundstrup, Reiner Rugulies

**Affiliations:** 1 National Research Centre for the Working Environment, Copenhagen, Denmark; 2 Department of Health Science and Technology, Aalborg University, Aalborg, Denmark; 3 Section of Epidemiology, Department of Public Health, University of Copenhagen, Copenhagen, Denmark

**Keywords:** absenteeism, cluster analysis, occupational exposure, psychosocial

## Abstract

**Objective:**

This study aimed to investigate the importance of combined psychosocial work factors for the risk of long-term sickness absence (LTSA).

**Methods:**

We followed 69 371 employees in the general working population (Work Environment and Health in Denmark study 2012–2018), without LTSA during the preceding year, for up to two years in the Danish Register for Evaluation of Marginalization. Using k-means cluster analyses and weighted Cox-regression controlling for age, gender, survey year, education, health-behaviors, and physical work demands, we determined the prospective association of 11 identified clusters – based on the combination of nine psychosocial work factors (recognition, quantitative demands, work pace, emotional demands, influence, justice, role clarity, role conflicts, and support from colleagues) – with the risk of LTSA.

**Results:**

During 124 045 person-years of follow-up, 6197 employees developed LTSA (weighted 8.5%). Using the cluster with the most favorable psychosocial scores as reference, clusters scoring poorly on several combined psychosocial factors had increased risk of LTSA. The cluster scoring poor on all nine psychosocial factors exhibited the highest risk [hazard ratio (HR) 1.68, 95% confidence interval (CI) 1.45–1.94]. Scoring poorly on one or two psychosocial factors did not increase the risk of LTSA when combined with favorable scores on the other psychosocial factors. Interaction analyses showed that gender, but not age and education, modified the association between cluster and LTSA.

**Conclusion:**

Scoring poorly on several combined psychosocial work factors plays an important role in the risk of LTSA. Scoring favorably on several psychosocial factors outweighed the potentially adverse effects of scoring poorly on one or two factors.

In most countries, the employer is responsible for ensuring a healthy and safe work environment. During the past century, preventive efforts have mainly focused on ’classical problems’ such as physical and chemical risk factors at the workplace. However, within the last decades, illness and absence from work due to mental health issues have led to increased focus on psychosocial factors within the work environment.

To this end, researchers have analyzed associations between the psychosocial work environment and health outcomes using different approaches. One approach is to define adverse psychosocial working conditions in accordance with theoretical models. The most frequently used – and best-evaluated – is the so-called “job strain model”, which is based on the notion that high job demands combined with low job control increase risk of diseases and disorders (1). Another widely-used model is the one related to effort–reward imbalance (ERI). This is based on the notion that an imbalance between high efforts spent at work and low rewards received, in terms of salary, recognition from the management, job security and promotion process, is health-hazardous (2). Likewise, researchers have also used the model of organizational justice, theorizing that exposure to low justice at work increases the risk of poor health (3). Recently, these findings were summarized in a meta-review of 72 systematic reviews, which reported convincing evidence that exposure to job strain and ERI is prospectively associated with an increased risk of poor health (4), in particular cardiovascular disease (5, 6) and depressive disorders (7–9). Additionally, low organizational justice has also been associated with increased risk of poor health. However, compared to the literature on job strain and ERI, the evidence is based on fewer studies and is less robust (10, 11).

A second approach is to examine numerous individual psychosocial work environment factors, rather than relying on theoretical models (12, 13). Thus, several studies have investigated the influence of one psychosocial factor while controlling for others, or explored their possible interactions. Such an approach raises the question whether specific combinations of risk factors may lead to synergistic or antagonistic effects. This question is difficult to answer using traditional interaction analyses, as the number of possible combinations grow exponentially, thereby substantially reducing statistical power.

A third, but rarely utilized, approach in work environment research is grouping individuals most alike in terms of the exposure variables in clusters in order to study the joint contribution of several psychosocial work factors. In the present paper, we utilize a clustering approach for examining possible joint associations between different combinations of psychosocial work factors and risk of poor health. Cluster analyses draw on hidden patterns in the data, and can – in the context of occupational research – identify clusters of workers with similar exposure characteristics. We have previously utilized this method to investigate the association between seven combined ergonomic work factors and risk of developing musculoskeletal pain (14).

In the present study, we used data from the Work Environment and Health in Denmark (WEHD) survey, which includes different psychosocial work environment variables. We included factors assumed to affect health outcomes via long-term stress processes, excluding factors such as workplace bullying or sexual harassment, as these presumably affect health and well-being more immediately. Likewise, we did not include factors at a higher hierarchical level, such as leadership quality, that may affect several of the other factors (15). Lastly, we excluded job insecurity, as it likely reflects the general economic- and labor-market situation more than the local work environment. Thus, we included the following nine factors: (i) quantitative demands (component of both job strain and ERI model, (ii) work pace (component of both job strain and ERI model), (iii) job control (component of the job strain model), (iv) recognition (component of ERI model), (v) justice at work (as a measure of organizational justice), (vi) emotional demands (a factor of increasing interest in epidemiological studies, in particular in Denmark (16), (vii) role clarity, (viii) role conflicts (two “classic” work environment factors, albeit with limited evidence from large-scale epidemiological cohort studies) (17) and (ix) collaboration and support from colleagues (representing a “classic” psychosocial work environment factor, which is also a part of the iso-strain model) (18) as well as a key component of the more recent approach of workplace social capital (19).

In the present analyses, we use long-term sickness absence (LTSA) as the outcome. LTSA, based on national registers, is strongly associated with measures of morbidity and mortality, and is therefore a reliable global indicator of poor health (20–22). LTSA constitutes a considerable burden for public finances in numerous countries, including Denmark, where municipalities reimburse employers for sickness absence benefits. We hypothesize that workers exposed to multiple risk factors exhibit a higher risk of LTSA compared to those exposed only to a few. However, as cluster analyses are inherently exploratory, we have no *a priori* assumption about which specific combinations most influentially increase the risk of LTSA. Against this background, the aim of this study is to investigate the importance of combined factors in the psychosocial work environment for the risk of LTSA. Furthermore, we explore whether age, gender and education modify the associations, as previous studies have suggested such effect modification (23–25).

## Methods

### Study design and population

This study combines all four waves (2012, 2014, 2016, and 2018) of the WEHD (26, 27) with the Danish Register for Evaluation of Marginalisation (DREAM). In each WEHD wave, probability samples of Danish residents aged 18–64 years, employed for a minimum of 35 hours per month with an income of at least 3000 DKK (approximately €400) per month in the past three months, were invited to participate. From 2012 to 2018, 228 173 people were invited, of which 127 882 (56%) responded to the survey. We included only people confirming through the survey that they were currently employed wage earners (N=110 357), ie, we did not include those self-employed. For people participating in more than one WEHD wave, we included only first occasion responses (N=73 298). Finally, we included only wage earners without LTSA during 52 weeks before their individual survey response and those replying to all questions about psychosocial work factors (N=69 371). Reporting is in accordance with the STROBE guidelines on cohort studies (28).

### Psychosocial work factors (exposure)

The psychosocial work factors included in WEHD are primarily based on the Copenhagen Psychosocial Questionnaire (COPSOQ). We included the following nine psychosocial work factors (15, 29–31):

• *PS1: – Recognition (REC) (1 item)*: How often is your work recognized and appreciated by management?

• *PS2: Quantitative demands (QUD) (5 items, Cronbach’s alpha 0.74)*: (i) How often do you have enough time for your work tasks? (Reversed in the normalized score.) (ii) How often do you experience deadlines that are difficult to keep? (iii) How often do you receive unexpected work tasks that put you under time pressure? (iv) How often are you available outside normal working hours? (v) How often do you have to work overtime?

• *PS3. Work pace (WOP) (1 item)*: How often is it necessary to keep a high work pace?

• *PS4. Emotional demands (EMD) (1 item)*: How often are you emotionally affected by your work?

• *PS5. Job control (JCO), also called “influence at work” or “decision latitude” (2 items, Cronbach’s alpha 0.75)*: (i) How often do you influence how you solve your work tasks? (ii) How often do you influence when you solve your work tasks?

• *PS6. Justice (JUS) (2 items, Cronbach’s alpha 0.73)*: (i) How often are all employees affected by a decision heard? (ii) How often are all the employees treated fair at the workplace?

• *PS7. Role clarity (RCL) (3 items, Cronbach’s alpha 0.77)*: (i) How often do you get the necessary information for doing your work? (ii) How often do you get the necessary help and instructions for doing your work? (iii) How often do you know exactly what your work task are?

• *PS8. Role conflicts (RCO) (1 item)*: How often do you experience contradictory demands at work?

• *PS9. Collaboration and support from colleagues (COL) (2 items, Cronbach’s alpha 0.77)*: (i) How often do you and your colleagues help each other in achieving the best possible results? (ii) How often do you and your colleagues collaborate when facing problems that require a solution?

Participants responded on a 5-point Likert scale ranging from ’always’ to ’never’. Responses were normalized on a scale ranging from 0–100, where never=0 and always=100 (32), except for the first quantitative demands item, which was reversed. The nine normalized scales were used for the cluster analysis.

### Long-term sickness absence (outcome)

We linked survey responses from the WEHD study to the DREAM register through a unique personal identification number from the Central Person Register that is provided to all Danish residents at birth and to foreigners when immigrating to Denmark (21, 22). In Denmark, the first 30 days of sickness absence are financially covered by the employer, after which the municipality can reimburse the remaining days. DREAM contains weekly – and not daily – information about reimbursement of sickness absence payments. Thus, ≥30 days of consecutive sickness absence corresponds to 6 consecutive weekly registrations in DREAM as the first week of sickness absence may begin on the last day of the week, and the last week of sickness absence may begin on the first day of the week (ie, 1 + 4 × 7 + 1 days = 30 days). Therefore, we defined LTSA as having registered sickness absence in DREAM for a period of ≥6 consecutive weeks for a period of up to 2 years, starting the week after replying to the survey (33). For the last WEHD wave (2018), the follow-up period is limited to about 1.5 years (until the end of 2019, ie, before the start of the COVID-19 pandemic).

### Control variables

Age (continuous variable) and gender (man, woman) for each individual were drawn from the Central Person Register of Denmark. Year of survey reply was a categorical variable (2012, 2014, 2016, and 2018). Highest completed education was drawn from a national register and included as a categorical variable (less than higher education, higher education). Health-behaviors included smoking status (categorical variable: daily, once in a while, ex-smoker, never), body mass index (BMI, kg/m^2^, continuous variable calculated from weight and height of the participants), leisure-time physical activity (continuous variable, total weekly hours of leisure physical activity). Physical workload (ergonomic index) was included as a continuous variable (33). Depressive symptoms [Major Depression Inventory (MDI), scale 0–50] was entered as a continuous variable (34). Frequency of musculoskeletal pain during the last three months was entered as a categorical variable (ie, daily, weekly, monthly, a few times, not at all). As health behaviors, depressive symptoms and frequency of musculoskeletal pain may also be potential mediators that could lead to over-adjustment, we present both minimally- and fully adjusted statistical models as well as sensitivity analyses.

### Statistical analyses

Using k-means cluster analyses (Proc FastClus, SAS version 9.4, SAS Institute, Cary, NC, USA) of the nine psychosocial work factors, we identified naturally occurring clusters in the working population (14). Checking for multicollinearity (r ≥ 0.70) did not lead to exclusion of any of the nine psychosocial factors. To determine the optimal number of clusters, we repeated the FastClus procedure with up to 20 clusters and compared the cubic clustering criterion (CCC), pseudo F, and explained variance (R^2^) against the number of clusters. This showed local peaks in CCC values (indicating possible good clustering) at 11, 13, 15 and 18 clusters with CCC values of 115, 113, 112 and 113, respectively. The corresponding pseudo F values were 6614, 5977, 5472 and 4908, respectively. The corresponding R^2^ values were 0.49, 0.51, 0.52 and 0.55, respectively. As all of these clustering possibilities could potentially be used, we chose the option with fewest clusters (ie, 11 clusters) to avoid a range of small clusters for further analyses.

Using the survey version of the Cox proportional hazard model (35) (Proc SurveyPhreg of SAS version 9.4.) we calculated hazard ratios (HR) of LTSA during follow-up for the different clusters. We used a time-to-first-event analysis and censored in case of one of the following criteria: Reaching the end of the two-year follow-up period, early retirement, disability pension, statutory retirement, emigration, or death, whichever came first. Each respondent was assigned a weight (based on information from national registers) to make the estimates representative. The weight variable repairs non-response and possible deviations of the probability sample from the population, and we did therefore not impute missing data.

We performed both minimally- and fully adjusted statistical models as well as sensitivity analyses. Model 1 (minimally adjusted) adjusted for age, gender, education, and year of survey reply. Model 2 (fully adjusted) additionally adjusted for health-behaviors and physical workload. Additionally, in three sensitivity analyses, we (i) controlled for musculoskeletal pain (pain frequency, categorical variable), (ii) controlled for mental health (MDI, continuous variable), and (iii) restricted the analyses to a subgroup of generally healthy individuals at baseline (excluding those with daily or weekly pain, or MDI scores ≥20). Finally, in the fully adjusted model 2, we tested for possible interactions of cluster with age, gender and education, respectively. In case of a statistical significant interaction (P<0.05), we provided additional stratified results. Results are reported as HR with 95% confidence intervals (CI).

## Results

**Figure 1:**
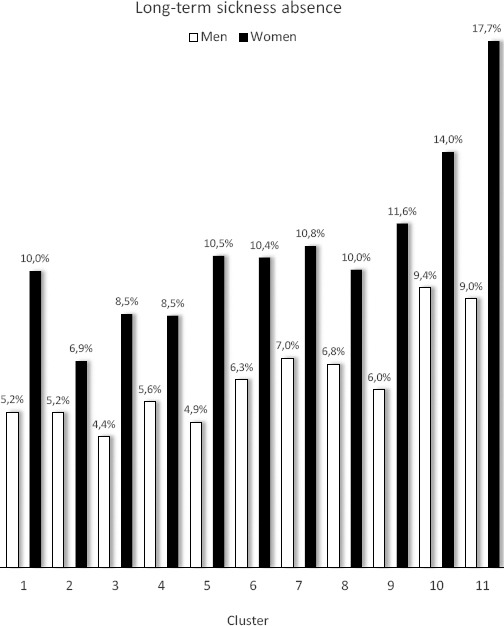
Unadjusted weighted percentages of long-term sickness absence during follow-up in the 11 identified clusters. Stratified for men and women.

Table 1 shows baseline characteristics of the 69 371 participants in terms of age, gender, education, health-behaviors, work characteristics, musculoskeletal pain and depressive symptoms. During 124 045 person-years of follow-up, 6197 employees developed LTSA (50 cases per 1000 person-years. The weighted percentage of LTSA during the follow-up period was 8.5%. Figure 1 shows the unadjusted weighted percentages of LTSA in each cluster, stratified by gender.

**Table 1:** Baseline characteristics of the participants (N=69 371). [WEHD=Work Environment and Health in Denmark; SD=standard deviation; EI=ergonomic index.]

	N	%	Mean	SD
WEHD wave				
2012	19 417	28.0		
2014	14 912	21.5		
2016	18 125	26.1		
2018	16 917	24.4		
Age (years)	69 371		45.9	10.8
Gender				
Men	32 856	47.4		
Women	36 515	52.6		
Highest education attained				
Less than higher education	37 711	54.7		
Higher education	31 216	45.3		
Body mass index (kg/m^2^)	67 406		25.7	4.4
Leisure-time physical activity (hours/week)	67 803		5.2	3.3
Smoking				
Yes, daily	9849	14.5		
Yes, once in a while	3515	5.2		
Ex-smoker	19 627	29.0		
No, never	34 812	51.3		
Physical work demands (EI 0-100)	67 655		19.0	16.4
Musculoskeletal pain last 3 months				
Daily	10 403	15.3		
Weekly	11 978	17.6		
Monthly	9580	14.1		
A few times	20 933	30.7		
Not at all	15 279	22.4		
Major Depression Inventory (0-50)	67 922		8.1	7.3

Table 2 shows the results for the 11 identified clusters. For each cluster, the weighted mean values of each of the nine psychosocial work factors (PS1–PS9) are presented and marked with color grades to ease interpretation. In other words, cluster 1 is characterized by favorable scores for all psychosocial factors. Cluster 2, 3, and 4, are characterized by poor scores for work pace, emotional demands, and recognition, respectively, but favorable scores for several of the other psychosocial factors. Cluster 5 is characterized by poor scores for role conflicts, emotional demands and moderate to poor for quantitative demands, but still having good scores for recognition, job control, justice, role clarity and support from colleagues. Cluster 6 is mixed with favorable scores for quantitative demands and work pace, while scoring moderate for the rest. None of these clusters (ie, cluster 2–6) showed increased risk of LTSA in any of the analyses, neither minimally nor fully adjusted or sensitivity analyses, compared with the reference group (cluster 1). Thus, scoring poorly on one or two psychosocial factors while having favorable scores on the other psychosocial factors was not associated with an increased risk of LTSA. However, individuals in clusters scoring poorly on several psychosocial factors had an increased risk of LTSA (cluster 7–11). Cluster 11, scoring poorly on all nine psychosocial factors, showed the highest risk (HR 1.68, 95% CI 1.45–1.94) in the fully adjusted analysis. Interaction analyses showed that gender (F=1.91, P=0.039), but not age and education, influenced the association between cluster and risk of LTSA. Consequently, we present gender-stratified analyses of model 2 in the last two columns of table 2.

**Table 2:** Weighted hazard ratios (HR) and 95% confidence intervals (CI) for the risk of long-term sickness absence during follow-up in the identified clusters (C) compared with the reference cluster (cluster 1, scoring overall best on the 9 different psychosocial work factors). For each cluster, the weighted mean values of each of the nine psychosocial work factors (PS1–PS9) are presented and marked with shades to ease the interpretation (Darker shades indicate worse scores). In Model 2, cluster and gender interacted, and the results are therefore presented stratified for gender in the last two columns. [REC=recognition; QUD=quantitative demands; WOP=work pace; EMD=emotional demands; JCO=job control (“influence at work”); JUS=justice; RCL=role clarity; RCO=role conflicts; COL=collaboration and support from colleagues.]

PS1REC	PS2QUD	PS3WOP	PS4EMD	PS5JCO	PS6JUS	PS7RCL	PS8RCO	PS9COL	Model 1 ^a^HR (95% CI)	Model 2 ^b^HR (95% CI)	Model 2 ^b^(women)
83	32	47	30	90	81	88	21	90	1	1	1
79	51	81	19	87	74	84	29	87	0.87 (0.73–1.03)	0.87 (0.73–1.04)	0.70 (0.55–0.89)
83	49	78	61	89	76	86	20	88	0.91 (0.78–1.07)	0.93 (0.79–1.09)	0.90 (0.75–1.09)
41	38	59	19	79	58	75	23	78	0.95 (0.80–1.12)	0.94 (0.79–1.11)	0.86 (0.69–1.08)
84	53	77	59	91	79	86	69	90	1.07 (0.91–1.25)	1.00 (0.85–1.18)	1.04 (0.86–1.27)
57	40	49	54	78	60	71	46	76	1.15 (0.99–1.34)	1.10 (0.94–1.29)	1.05 (0.87–1.27)
36	53	80	59	82	60	75	31	82	1.26 (1.09–1.46)	1.20 (1.03–1.39)	1.09 (0.91–1.31)
46	58	79	20	77	54	69	61	78	1.22 (1.03–1.43)	1.15 (0.98–1.36)	1.00 (0.80–1.25)
60	63	83	64	75	55	68	59	75	1.29 (1.12–1.49)	1.24 (1.07–1.43)	1.21 (1.02–1.44)
23	45	62	40	53	36	56	46	59	1.61 (1.39–1.87)	1.45 (1.25–1.69)	1.33 (1.10–1.62)
21	66	86	68	62	36	54	69	69	1.96 (1.70–2.26)	1.68 (1.45–1.94)	1.68 (1.41–2.01)

a Adjusted for age, gender, education, and year of survey reply.

b Model 1 + health-behaviors (body mass index, smoking, leisure physical activity) and physical workload (ergonomic index).

Table 3 shows the sensitivity analyses. Controlling for frequency of pain reduced the risk estimates slightly, while controlling for depressive symptoms resulted in loss of statistical significance for the estimates of all clusters. However, based on visual inspection of the point estimates and their 95% CI, cluster 10 and 11 still appear to have an increased risk for LTSA compared with cluster 2, 3 and 4 (no overlap of CI). Including only generally healthy individuals at baseline also reduced the estimates, leaving only cluster 11 with a statistically significantly increased risk. Further, the analyses including generally healthy individuals at baseline showed that cluster 2 (scoring favorable on 8 of the 9 psychosocial work factors with the exception of work pace) had a lower risk compared to the reference group (scoring favorable on all 9 psychosocial work factors).

**Table 3:** Weighted hazard ratios (HR) and 95% confidence intervals (CI) for the risk of long-term sickness absence in sensitivity analyses of model 2 (table 2).

C	Model 2 ^a^controlling for pain	Model 2 ^b^controlling for depressive symptoms	Model 2 ^c^only generally healthy individuals
1	1	1	1
2	0.86 (0.72–1.03)	0.85 (0.71–1.01)	0.78 (0.62–0.99)
3	0.89 (0.76–1.04)	0.87 (0.74–1.02)	0.81 (0.66–1.01)
4	0.90 (0.76–1.06)	0.88 (0.74–1.04)	0.82 (0.66–1.02)
5	0.98 (0.83–1.15)	0.93 (0.79–1.10)	1.00 (0.81–1.23)
6	1.04 (0.89–1.22)	0.97 (0.83–1.13)	0.99 (0.80–1.22)
7	1.10 (0.95–1.28)	1.04 (0.89–1.21)	0.97 (0.79–1.19)
8	1.07 (0.91–1.27)	1.02 (0.86–1.21)	1.02 (0.81–1.28)
9	1.11 (0.96–1.28)	0.99 (0.86–1.15)	1.11 (0.91–1.35)
10	1.29 (1.11–1.51)	1.16 (0.99–1.36)	1.17 (0.94–1.47)
11	1.42 (1.22–1.64)	1.15 (0.99–1.35)	1.31 (1.04–1.64)

a Controlled for musculoskeletal pain (pain frequency, categorical variable).

b Controlled for mental health (Major Depression Inventory, continuous variable).

c Estimates in a subgroup analysis including only generally healthy individuals at baseline (excluding those with daily or weekly pain and scoring ≥20 on MDI).

Table 4 shows the distribution of psychosocial work clusters for the different job groups in the study. Job group *per se* was not included as a variable in the statistical analyses but presented to put the results into context and examine whether the different clusters represented specific job groups. Overall, the clusters did not represent any particular job groups, ie, the different job groups included many different clusters, though some jobs had more high-risk clusters than others. Examples of job groups with relatively many individuals in high-risk clusters were police officers and prison guards, mail carriers, medical doctors, vocational education teachers, food and related products industrial laborers, social workers, and high school teachers. At the other end of the scale, examples of job groups with relatively few individuals in high-risk clusters were clinic and dental assistants, farmers and gardeners, building and cleaning supervisors, child daycare workers (note: taking care of children in the workers’ own home), as well as hairdressers and beauticians.

**Table 4:** Distribution of psychosocial work clusters in each job group (row percentages). Ranked in descending order from overall poorest to most favorable scores. Based on table 2, clusters 7–11 were clusters showing increased risk of LTSA in some of the analyses. Darker shades indicate higher percentage.

Job group	N	Cluster distribution (row percentage)										

		1	2	3	4	5	6	7	8	9	10	11
Police officers and prison guards	416	3	3	3	7	5	12	7	8	16	17	18
Mail carriers	365	4	8	5	8	8	6	5	13	11	18	14
Medical doctors	583	2	3	15	2	8	5	16	5	25	5	11
Vocational education teachers	557	4	2	10	6	6	14	13	4	15	9	17
Food and related products industrial labourers	696	6	10	3	13	7	9	6	12	6	20	8
Social workers	722	5	4	12	2	9	10	11	3	21	7	16
High school teachers	740	3	3	11	4	7	12	21	4	17	5	13
Nurses	2576	4	3	13	3	9	10	18	2	20	6	13
Travel attendants and conductors	155	6	5	4	8	8	10	13	9	6	14	16
Journalists	365	8	5	10	5	8	7	15	11	16	7	8
Teachers	2828	5	2	14	2	8	14	15	2	21	5	12
Special needs teachers	452	7	1	13	4	6	19	14	2	15	8	12
Fire-fighters and protective service professionals	304	8	3	4	9	8	18	6	6	8	22	7
Psychologists	258	6	4	10	5	10	11	16	2	22	3	10
Bus, taxi, and train drivers	616	11	3	5	16	5	11	7	6	4	23	9
Professors and researchers at universities	1067	6	7	12	6	8	10	12	7	20	3	11
Health and personal care workers	3475	8	3	12	4	12	11	14	4	14	7	12
Military personnel	435	7	8	4	7	11	15	4	13	15	7	8
Special educators	1198	8	2	11	5	10	20	12	2	14	6	10
Service workers	77	12	5	5	9	5	9	18	10	10	5	10
Social science academics	478	8	11	10	6	10	10	9	8	16	3	9
Customs inspectors and tax officials	429	6	6	8	12	8	11	8	11	13	11	6
Educated child care workers (nursery and kindergarten)	2191	7	3	14	3	12	13	15	2	17	5	8
Manual workers in health care (e.g. porters)	497	8	3	9	6	12	14	11	4	12	11	10
Sales and purchasing agents	1485	6	12	11	8	9	7	9	11	15	5	8
IT-technicians	476	8	10	9	11	7	10	6	12	13	7	8
Handicraft and precisions workers	188	8	9	7	16	5	8	8	8	7	12	11
Customer services clerks	645	8	6	11	10	12	9	10	8	10	9	8
Executive, medical, and legal secretaries	1553	7	10	13	8	7	10	11	8	12	7	7
Cashiers	411	9	11	7	9	11	7	8	9	9	10	11
Lawyers	889	5	12	11	8	7	9	10	12	16	5	6
Freight forwarders	1057	9	10	6	11	8	8	9	11	10	9	8
Smiths	1265	8	9	3	16	8	9	6	14	7	13	6
Auditors, advisors and analysts	2450	5	15	11	7	10	7	9	10	15	4	7
Butchers and bakers	194	5	14	7	9	14	8	6	12	5	8	11
Mechanics	605	10	10	5	13	7	9	7	16	8	9	8
Storage and transport labourers	731	11	7	5	15	10	8	6	13	7	13	7
Managers	3281	5	12	13	4	16	6	10	9	16	2	6
Truck drivers	734	15	11	5	14	6	7	3	12	4	18	7
Manufacturing labourers	555	10	12	4	13	7	8	7	11	7	15	6
Physiotherapists and occupational therapists	685	7	2	14	4	8	16	17	3	15	5	8
Pharmaconomists and bioanalysts	475	8	7	13	9	5	8	18	5	11	9	8
Accounting staff	1289	9	11	10	13	7	7	11	10	11	6	5
Cooks and waiters	408	7	14	12	7	12	3	9	12	11	6	8
Cleaners	1482	13	9	8	9	11	10	7	6	8	11	8
Science and engineering associates	1541	9	10	8	12	8	10	8	11	9	7	8
Machine operators	1732	10	8	5	13	8	10	7	11	8	14	6
Shop salespersons	1665	8	11	9	8	10	8	9	10	12	7	7
Mobile plant operators and drivers	254	9	11	4	14	5	8	8	14	9	14	3
Engineers and architects	2128	8	14	11	8	8	8	8	12	14	4	5
Librarians, archivists and curators	437	9	6	10	8	9	13	9	5	15	7	8
Electricians	797	10	8	4	17	6	11	5	15	6	11	6
Technical draftsmen	281	9	8	6	15	5	11	10	12	11	6	7
Assemblers	440	12	9	5	14	6	8	5	13	9	14	5
Accountants	720	9	13	7	12	7	9	12	9	10	6	6
Scientific academics	367	7	14	13	10	6	7	12	7	14	5	6
IT consultants	1649	9	13	8	10	7	8	7	12	14	5	6
Office staff and secretaries	3726	10	10	10	11	6	11	10	7	10	8	7
Construction workers	862	11	9	4	15	10	12	6	10	5	12	6
Teachers’ aides and pedagogical assistants	917	11	4	14	3	13	14	12	2	11	7	7
Painters	265	9	12	6	12	9	11	5	10	6	11	7
Food preparation assistants	498	11	12	11	7	13	8	9	10	7	7	6
Pharmacists, dentists and veterinarians	311	6	14	19	6	7	7	10	6	14	2	8
Bricklayers and plumbers	722	12	12	5	12	7	9	6	16	7	9	6
Laboratory technician	450	12	9	13	14	2	8	9	10	10	7	7
Carpenters and woodworkers	582	8	15	5	14	8	8	7	14	7	10	3
Manual work without specification	178	13	8	8	15	5	9	7	6	7	15	6
Concrete workers	473	12	10	4	13	8	14	4	12	5	12	7
Health care workers without specification	311	11	7	15	8	11	9	14	5	11	4	6
Clinic and dental assistants	361	10	6	15	9	9	12	13	6	6	8	6
Farmers and gardeners	398	13	9	6	9	12	13	9	10	8	7	6
Building and cleaning supervisors	610	17	5	8	10	11	16	5	6	6	8	6
Child daycare workers	643	13	2	13	5	11	28	8	1	6	6	7
Hairdressers and beauticians	80	10	7	16	9	21	9	14	.	9	.	5

## Discussion

In the present study, we used k-means cluster analyses to identify clusters of workers with similar exposure characteristics. The analysis identified 11 different clusters corresponding to different combinations of psychosocial work exposures. Our study showed that scoring poorly on several psychosocial work factors plays an important role in the development of poor health, expressed herein as LTSA. Importantly, scoring favorable on several psychosocial factors outweighed the potentially adverse effects of scoring poorly on one or two factors.

Karasek’s job-strain model is probably the most thoroughly tested and documented model in the field of psychosocial work environment research (5–8, 36, 37). The job-strain model states that a combination of high demands and low control has detrimental effects on employees’ health. In the present study, cluster 11 corresponds to high demands and low control and confirms the adverse effect of this combination on employees’ health. However, this cluster was also characterized by poor scores on all the other psychosocial work factors. None of the clusters came out with the combination of high demands and low control while simultaneously scoring favorable, or even moderate, on other psychosocial work factors. Thus, the strong and consistent association between high job-strain and poor health reported in the literature may partly be due to co-occurrence with several other adverse psychosocial work factors.

Siegrist’s ERI model is another well-known framework, proposing that a certain combination of psychosocial work exposures increases the risk of poor health (6, 9, 36). In cluster 4 and 10, poor scores on recognition – which can be considered as a type of low reward – were combined with favorable scores on quantitative demands and work pace (effort). This combination should, according to Siegrist’s model, not increase the risk of adverse health outcomes. While this was true for cluster 4, we found increased risk of LTSA in cluster 10. The main difference between these two clusters is the co-occurrence of several adverse psychosocial exposures in cluster 10, ie, poor scores on job control, justice, role conflicts and support from colleagues. Hence, these negative factors appear to outweigh the potential benefit of low efforts.

Collectively, our results are broadly in line with these known theoretical models, ie, job-strain and ERI. However, our study also revealed the relevance of identifying naturally occurring clusters of psychosocial work exposures, as these represent common combinations of workplace exposures.

We assumed *a priori* that the cluster with the most favorable psychosocial work scores (cluster 1) should be defined as the reference cluster – ie, cluster 1 had high scores for recognition, job control, justice, role clarity and collaboration combined with low scores for quantitative demands, work pace, emotional demands and role conflicts. Nevertheless, clusters 2, 3, 4, 5 and 6 did not – in any of the analyses – show increased risk of LTSA and may even suggest a J-shaped association. In the gender-stratified analysis (women) and in the sensitivity analysis including all generally healthy workers at baseline, cluster 2 (and a tendency for cluster 3 and 4) even showed lower risk of LTSA compared with cluster 1. Thus, a J-shaped association may exist between combined psychosocial work factors and risk of LTSA, as also indicated by the unadjusted data in figure 1. Thus, having one psychosocial ’challenge’ – in the present case in terms of higher work pace (cluster 2), higher emotional demands (cluster 3) or lower recognition (cluster 4) – may be better than having no challenges at all, as long as the majority of the other psychosocial work factors are favorable. However, the J-shape may also be due to selection, as it is conceivable that more resilient employee chooses more challenging job tasks. While future studies should explore this J-shaped association in more detail, the present results suggest that scoring favorably on several psychosocial factors may outweigh the potentially adverse effects of scoring poorly on one or two factors. This has important practical implications, ie, workplaces may focus on aspects of the psychosocial work environment that are feasible to improve. For example, if emotional demands are difficult to reduce because of the inherent working conditions of dealing with patients and clients, sufficient recognition for the work, ensuring a high level of influence, securing collegial support, making the different roles clear, may be a way to reduce the risk of LTSA.

Although some of the nine included work environment factors may be more important than others, we treated all psychosocial work factors equally and did not weigh if the factor was about a potential stressor, (such as high emotional demands) or about a potential or lack of resource (such as collaboration and support from colleagues). The present data do not show a clear pattern indicating one or two “super factors” for the risk of LTSA, but rather an accumulative effect of more adverse factors increasing this risk. However, this should be replicated in future studies specifically designed to test this hypothesis.

Finally, interaction analyses suggest that gender, but not age and education, influenced the association between cluster and risk of LTSA. The most pronounced gender differences were that women (but not men) in cluster 2 – high work pace, but favorable scores on the other factors – had reduced risk of LTSA, while men (but not women) in clusters 7 and 8 – mixed scores of poor, moderate and favorable on the different psychosocial work factors – had increased risk of LTSA. As the labor market in Denmark is somewhat gender-segregated, eg, relatively more women in care work and more men in construction work, job-group specific clusters may be speculated to influence these findings. However, judging by the distribution of clusters within each job group (table 4), no clear indication of this exists. While the present study does not explain the cause of these gender-differences, future studies should be cautious of testing for possible gender-interactions.

### Limitations and strengths

We covered a large spectrum of the psychosocial work environment, by including nine different psychosocial work factors yielding 11 different clusters. However, we cannot claim the list of psychosocial factors to be exhaustive. The Danish Psychosocial Work Environment Questionnaire (DPQ) included 38 different psychosocial work factors (12). Several of these factors, eg, cognitive demands, were not measured in WEHD. Other factors were measured, but we decided not to include them in the analyses for the reasons explained in the introduction. Thus, data availability and assumption-based decisions limit the selection of work factors included in the present analysis.

Although we included factors related to theoretical models (eg, recognition as a part of the ERI model), our study was rather exploratory than theory driven. For example, we refrained from dichotomizing the 11 factors into demands versus resources, as suggested by the job-demands resources theory (38). Instead, we took a data-driven approach in identifying clusters most strongly associated with LTSA. We acknowledge that a more theory-driven approach would have likely resulted in different clusters. In addition, we measured four of the nine work factors with a single item only, which may have limited content validity. We would have preferred validated multi-item scales, like in the DPQ, but many of these were not available in WEHD. As the exposure variables on psychosocial work factors were self-reported, and we therefore chose an objective outcome measure, register-based LTSA, to avoid common-method biases. Nevertheless, some of the control variables were self-reported, e.g. mental health. As the state of mind may influence ratings of both psychosocial work factors and mental health, some misclassification bias may occur, ie, those with poor mental health may – everything else being equal – be more likely to rate the psychosocial work factors as worse. However, it may also be that poor working conditions had already led to poor mental health which in turn led to LTSA (mediation), and controlling the analyses for mental health may therefore have led to over-adjustment. In fact, poor mental health may act both as a confounder and mediator. For this reason, we also performed sensitivity analyses including only generally healthy individuals at baseline, which confirmed that the combination of many adverse psychosocial work factors (cluster 11) is associated with increased risk of LTSA. We did not include offending behaviors, such as workplace bullying, sexual harassment or violence, in the selection of cluster variables, because we did not want to mix factors that may have an immediate effect on health with other factors that may have a more long-term effect on health. This may pose a limitation because some of the highly unfavorable clusters could, speculatively, be clusters with a high prevalence of offending behaviors. The possibility also exists that factors at a higher hierarchical level, eg, leadership quality and behavior of the immediate supervisor or the top management, may have influenced several of the included factors. Further research should examine these aspects. Likewise, to circumvent some of the methodological limitations of the present study, future studies may consider using job-exposure matrices of clustering of psychosocial work factors as exposure variable and use diagnosed poor mental health obtained from registers (eg, depression) as a mediating factor in the risk of register-based absence from work or dropout from the labor market. However, such an approach is not without challenges, eg, register-based depression does not include undiagnosed cases. Furthermore, based on the distribution of clusters within job-groups (table 4), there may only be limited potential in constructing job-exposure matrices. Thus, job group differences for psychosocial work factors are not nearly as clear as those reported previously for physical workload (33). Using register-based LTSA as outcome also has some limitations. In Denmark, the cause of sickness absence is not registered (due to law). Thus, sickness absence from work can have several causes, including both work-related and non-work related diseases. In addition, we did not analyze short-term sickness absence or turnover. Nevertheless, LTSA is a good proxy of overall health that is strongly associated with adverse health endpoints in terms of disability pension and mortality (20). To ensure generalizable findings, the sample should be large and representative. In the present study, we used random samples of wage earners drawn over four different time points from 2012 to 2018. Further, we used model-assisted weight to ensure that estimates were representative. This strengthens the generalizability of the findings.

### Concluding remarks

The combination of scoring poor on several psychosocial work factors plays an important role in the development of poor health in terms of LTSA. Importantly, scoring favorable on several psychosocial factors outweighed potentially adverse effects of scoring poor on one or two factors. This knowledge is of practical relevance especially for workplaces where the inherent conditions of the work make it difficult to improve all psychosocial work factors.

### Data availability statement

The authors encourage collaboration and use of the data by other researchers. Data is stored on the secure server of Statistics Denmark, and researchers interested in using the data for scientific purposes should contact the authors.

### Funding

Professor Lars L Andersen obtained a grant from the The Danish Working Environment Research Fund for this project (Arbejdsmiljøforskningsfonden, grant number 20195100758). The funder had no role in study design, data collection, data analysis, data interpretation, or writing of the paper.

### Conflicts of interest

The authors declare no conflicts of interest
